# I Never Thought About Leaving: Why Residents Aged in Place Within Neighborhoods Experiencing Urban Decline

**DOI:** 10.1093/geroni/igaa057.1562

**Published:** 2020-12-16

**Authors:** Kaitlyn Langendoerfer

**Affiliations:** Case Western Reserve University, Honesdale, Pennsylvania, United States

## Abstract

Despite the rich literature on “aging in place”, few studies have examined why older adults remain within neighborhoods that have experienced urban decline. This study defines “aging in place” as the process of aging within one’s neighborhood over the life course. Further, much of the literature surrounding disadvantaged, urban communities have concentrated on those who left and portray those who remain as trapped. This is particularly true for older African Americans. The purpose of this study was to understand what influenced older, African American residents to stay within their declining neighborhoods. Data was utilized from 4 years of ethnographic observations with 30 older (age 60+), African-American adults who aged within Cleveland. Additionally, multiple in-depth life history interviews were conducted with 13 of these residents. Data was analyzed using grounded theory techniques. All residents expressed a desire to remain within their neighborhoods and many indicated that they never thought about leaving. While each resident had their own reasons for staying, common themes emerged related to: 1) autobiographical insideness, 2) sense of identity, 3) home ownership, and 4) interdependent lives. This study has important implications for research related to aging in place and place attachment. For one, it provides a counter narrative to the dominant notion that residents of poor neighborhoods would leave if they had the resources to relocate. Additionally, by using a narrative method, the older adults were able to explain for themselves why they stayed and describe the meaningful lives they created despite neighborhood decline.

